# Ulcerative colitis: functional analysis of the in-depth proteome

**DOI:** 10.1186/s12014-019-9224-6

**Published:** 2019-01-29

**Authors:** Armin Schniers, Rasmus Goll, Yvonne Pasing, Sveinung Wergeland Sørbye, Jon Florholmen, Terkel Hansen

**Affiliations:** 1 Natural Products and Medicinal Chemistry Research Group, Department of Pharmacy, Faculty of Health Sciences, UiT The Arctic University of Norway, 9037 Tromsø, Norway; 2 Department of Medical Gastroenterology, University Hospital of North Norway, Tromsø, Norway; 3 Gastroenterology and Nutrition Research Group, Department of Clinical Medicine, Faculty of Health Sciences, UiT The Arctic University of Norway, Tromsø, Norway; 4 Division of Internal Medicine, University Hospital of North Norway, Tromsø, Norway; 5 Department of Clinical Pathology, University Hospital of North Norway, Tromsø, Norway

**Keywords:** Inflammatory bowel disease, Ulcerative colitis, Calprotectin, Signal peptidase complex

## Abstract

**Background:**

Ulcerative colitis (UC) is one major form of inflammatory bowel disease. The cause and the pathophysiology of the disease are not fully understood and we therefor aim in this study to identify important pathophysiological features in UC from proteomics data.

**Methods:**

Colon mucosa biopsies from inflamed tissue of untreated UC patients at diagnosis and from healthy controls were obtained during colonoscopy. Quantitative protein data was acquired by bottom-up proteomics and furthermore processed with MaxQuant. The quantitative proteome data was analyzed with Perseus and enrichment data was analyzed by ClueGO for Cytoscape.

**Results:**

The generated proteome dataset is to-date the deepest from colon mucosa biopsies with 8562 identified proteins whereof 6818 were quantified in > 70% of the samples. We report abundance differences between UC and healthy controls and the respective p values for all quantified proteins in the supporting information. From this data set enrichment analysis revealed decreased protein abundances in UC for metallothioneins, PPAR-inducible proteins, fibrillar collagens and proteins involved in bile acid transport as well as metabolic functions of nutrients, energy, steroids, xenobiotics and carbonate. On the other hand increased abundances were enriched in immune response and protein processing in the endoplasmic reticulum, e.g. unfolded protein response and signal peptidase complex proteins.

**Conclusions:**

This explorative study describes the most affected functions in UC tissue. Our results complemented previous findings substantially. Decreased abundances of signal peptidase complex proteins in UC are a new discovery.

**Electronic supplementary material:**

The online version of this article (10.1186/s12014-019-9224-6) contains supplementary material, which is available to authorized users.

## Background

Ulcerative colitis (UC) is a form of inflammatory bowel disease (IBD). The chronic inflammation of the colon in UC starts at the rectum and can progress continuously to proximal colon parts. UC affects 0.51% of the European and 0.25% of the North American population, with increasing prevalence [1]. The disease has a complex pathophysiology and the exact disease causes remain unclear. A genetic component, environmental factors, defects of the epithelial barrier, and dysregulated immune responses are involved [2].

Pathophysiological changes likely lie or are reflected in the abundance and state of proteins, the major functional units in every tissue. The proteomic analysis of colon biopsies affected by UC gives data on these changes at the main site of the disease. Advances in sample preparation, instrumentation, and analysis software allow for increasing proteome coverage and improved quantification. Higher proteome coverage, growing and new pathway databases, and improved software packages benefit comprehensive enrichment analyses of tissues.

Proteomic studies with different designs regarding samples and analytical method were previously conducted on colon mucosa affected by UC. Inflamed mucosa from untreated patients at UC debut [3] as well as macroscopically normal mucosa from treated UC patients [4] have been compared to biopsies from healthy controls in previous studies. A comparison of inflamed mucosa from untreated UC patients with mucosa from healthy controls as well as from Crohn’s disease patients was conducted on pediatric patients [5]. Another study compared inflamed and non-inflamed tissue from the same patients [6]. Despite the differences in the study designs, the studies present remarkably similar proteome changes [3]. They revealed novel insights into the UC pathophysiology, e.g. the role of neutrophil extracellular traps [4] and S100 proteins [3]. They also identified biomarkers for differentiating between UC and Crohn’s disease [5] and between pancolitis and partial colitis [7]. Furthermore, multiple studies investigated specific proteins of interest and their functions in UC, such as inducible nitric oxide synthase (NOS2) [8] and histaminase (AOC1) [9].

However, despite the challenges in extracting biological information from omics data and the shortcomings of focusing on proteins with the highest fold-changes [10], enrichment analyses were not the main scope of previous proteomics studies on UC. Omics studies typically produce large amounts of data. Especially in experiments with strong phenotypes these datasets can contain so many significant findings that a sensible interpretation without additional statistical analyses is barely possible. Interpretations based on only the highest fold-changes in such a dataset disregard large amounts of data and fail to recognize functional changes as a result of multiple moderately different abundances. Moreover, the biological interpretation is prone to be arbitrary, and it has further disadvantages. Enrichment analyses can overcome these difficulties [10] and the enrichment factors give an additional indication for the importance of the respective functions for the disease pathophysiology.

Beside proteomics, transcriptomic approaches have been applied to investigate UC [11]. These studies show differential mRNA expressions in UC tissue. The pathophysiological implications of these finding however are less clear because the correlation of mRNA and protein abundances is poor. The cause for that low correlation is that both the translation and the degradation of proteins are subject to several regulation mechanisms that are independent of mRNA levels [12].

We conducted a comprehensive enrichment analysis on a proteomics dataset of 8562 identified proteins from colon mucosa biopsies.

## Materials and methods

### Patients included and biopsies collection

Mucosal biopsies were collected from newly diagnosed treatment-naïve UC patients in Norway. The UC diagnosis was established upon clinical, endoscopic and histological criteria defined by the European Crohn and Colitis Organization (ECCO) guidelines [13]. Furthermore, the degree of inflammation was evaluated during colonoscopy using the scoring system of ulcerative colitis disease activity index (UCDAI) [14]. Moreover, TNF mRNA levels were measured by real-time PCR to assess the level of UC activity [15]. Subjects admitted for a cancer screening and with normal colonoscopy and histological findings served as healthy controls. None of the recruited subjects suffered from irritable bowel disease and they were not on nonsteroidal anti-inflammatory drug medication prior to the colonoscopy. The biopsies from the UC patients were obtained from the most inflamed area in rectum or sigmoid colon. Biopsies from the control group were obtained from the rectum. From each study participant, 3 adjacent biopsies were obtained from the inflamed mucosa. One biopsy was immediately immersed in RNAlater (Qiagen, Germany). The second biopsy was frozen immediately in a dry cryotube tube at − 70 °C until further analysis. The third biopsy was obtained for ordinary histological examinations (haematoxylin and eosin staining). 17 patients with debut of UC and 15 healthy controls were recruited as shown in Table 1.

**Table 1:** Baseline characteristics in patients included at debut of ulcerative colitis and in healthy controls (see “Patients included and biopsies collection” and “Baseline characteristics” sections for further details)

Study group	Number of subjects	Average age (SD)	Sex female/male	Median TNF-α (IQR)	Average UCDAI score (SD)	Average Geboes index (SD)	Extend (Montreal classification [65])
UC patients	17	39.4 (16.7)	4/13	14,350 (16,725)	8.7 (2.2)	7.9 (3.8)	2 Proctitis 9 left sided UC 6 Extensive UC
Healthy control	15	51.9 (14.3)	5/10	4500 (2400)	–	–	–

UCDAI score and Geboes index averages calculated from available data for 15 and 16 patients, respectively

### Baseline characteristics

The baseline characteristics are shown in Table 1 (see Additional file 1: Table 1). All subjects included showed typical histological findings according to UC and normal, non-inflamed mucosa, respectively.

### Sample preparation

Samples were homogenized with 250 µl cooled lysis buffer [8 M urea, 5% sodium deoxycholate (SDC), 100 mM triethylammonium bicarbonate buffer pH 8.5 (TEAB)] in MagNA Lyser Green Bead tubes (Roche Diagnostics AG, Rotkreuz, Switzerland) with a MagNA Lyser Instrument (Roche Diagnostics AG, Rotkreuz, Switzerland) for 35 s at 6500 rpm. Lysates were frozen at − 70 °C until further sample preparation. All samples and standards were diluted/produced to contain 5 mM TEAB for the BCA assay (Pierce™ BCA Protein Assay Kit, Thermo Fisher Scientific). The assay was performed according to the manufacturer’s protocol. Lysate aliquots of 60 µg protein were transferred to Protein LoBind tubes (Eppendorf AG, Hamburg, Germany). Disulfide bridges were reduced with 1,4-dithiothreitol (DTT) at a concentration of 5 mM by incubation at 54 °C for 30 min. Cysteins were alkylated with 15 mM iodoacetamide (IAA) and incubation for 30 min at room temperature in the dark. DTT solution corresponding to a final concentration of 5 mM was added to remove excess IAA. Lys-C predigestion was performed under gentle agitation for 8 h at 37 °C with 0.6 µg Lys-C (enzyme-to-protein ratio 1:100, w/w, Wako Chemicals GmbH, Neuss, Germany) in a buffer containing 1 mM calcium chloride, 6 M urea, and 100 mM TEAB. Calcium chloride solution, 3 µg trypsin (enzyme-to-protein ratio 1:20, w/w, Sequencing Grade Modified Trypsin, Promega Corporation, Madison, USA), water, and 1 M TEAB were added to a final concentration of 1 mM calcium chloride, 1 M urea, and 100 mM TEAB. Tryptic digestion was performed under gentle agitation for 16 h at 37 °C.

Equal peptide amounts from each sample were combined to produce a standard mixture, which was subsequently labelled with TMT126. Aliquots corresponding to 25 µg peptides were labelled with the remaining TMTsixplex isotopes according to the manufacturer’s protocol. 0.4 mg TMT reagent were used for 25 µg peptides from samples with starting protein concentrations ≥ 2200 µg/ml. Higher amounts of TMT reagent were used for lower starting concentrations (see Additional file 1: Table 2).

The differently labelled peptides were mixed in equal amounts to in total 100 µg peptides. The digests were acidified with 50% formic acid (FA) to a final concentration of 2.5% FA and pH ≤ 2. The samples were centrifuged at 16,000 g for 15 min and the supernatants were carefully transferred to fresh tubes. Acetonitrile was removed by evaporation in a vacuum concentrator.

Trifluoroacetic acid (TFA) was added to a concentration of 0.1%. The samples were fractionated with Pierce™ High pH Reversed-Phase Peptide Fractionation Kit (Thermo Fisher, Rockford, USA) according to the manufacturer’s protocol. Samples exceeding 300 µl were stepwise loaded with 2 min centrifugation after each loading. The fractions were dried in a vacuum concentrator and redissolved in 10 µl 0.1% TFA for subsequent LC–MS/MS analyses.

### LC–MS/MS

The nano-LC–MS/MS analysis was performed on a Q Exactive mass spectrometer coupled to an EASY-nLC 1000 system (Thermo Fisher Scientific, Bremen, Germany).

Shortly, 2.0 µg peptides, as measured with a Nanodrop 2000 (Thermo Fisher Scientific, Bremen, Germany) at a wavelength of 205 nm and extinction coefficient 31 mg/ml [16], per sample were injected. They were concentrated on a reversed-phase trap column and subsequently separated on a reversed-phase main column. A binary solvent gradient of 0.1% FA (solvent A) and 0.1% FA in acetonitrile (solvent B) was used. The ACN proportion was increased from 0 to 5% over 19 min, further to 30% at 180 min and to 100% at 200 min. A detailed description of the LC–MS/MS conditions can be found in the supporting information (see Additional file 1).

### Data evaluation

The raw data was analyzed with MaxQuant against a UniProt fasta file of all human proteins (see Additional file 1) [17, 18]. Further analyses were performed with Perseus [19] and Cytoscape/ClueGO [20, 21].

The data processing and visualization in Perseus is partially based on a recently published protocol by the Perseus developers [22]. The intensities were log2-transformed in Perseus, and the entries were filtered for the labels “potential contaminant”, “reverse” and “only identified by site”. Standard intensities were subtracted from the corresponding sample intensities and the resulting intensities *Z*-score transformed (matrix access: columns), which returns the normalized values (see Additional file 1).

### Statistics and generation of figures and lists

Statistical analyses were performed in Perseus. The samples were assigned to the groups UC patients and healthy controls, respectively, and the groups containing the separate values for each sample were compared to each other with a two-sample test. Proteins were annotated with gene ontology terms from the databases GOBP (biological process), GOMF (molecular function), GOCC (cellular component) [23], KEGG [24], GSEA [25], UniProt keywords [18], InterPro [26], Reactome [27], and PROSITE [28]. The lists and the article figures were prepared in Perseus and the Cytoscape app ClueGO (see Additional file 1). The WikiPathways database was used for the ClueGO analysis [29]. Pathways were mapped with HumanCyc (see Additional file 1) [30].

## Results and discussion

### Exploratory analysis

**Fig. 1:**
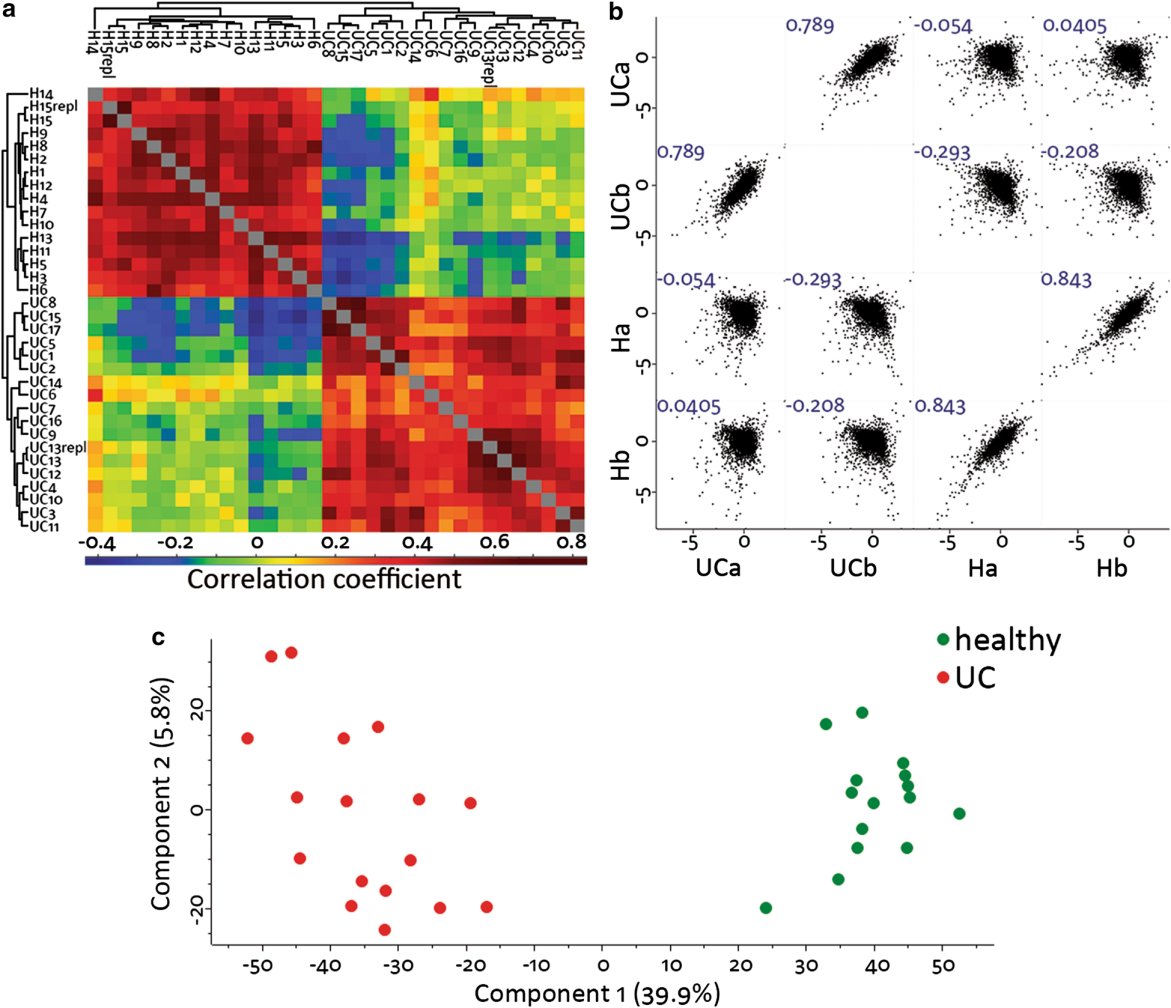
Exploratory analysis. **a** Hierarchical clustering of the correlation coefficients of 6829 protein intensities (proteins quantified in at least 70% of the samples) after normalization shows a clear separation between ulcerative colitis (UC) and healthy controls. The two replicates (labeled with repl) show the highest correlations. **b** Multi-scatter plot of the averaged profiles for UC2, 3, 4, 6, 7, 11, 13, 14, and 16 (UC a), UC1, 5, 8, 9, 10, 12, 15, and 17 (UC b), H3, 4, 5, 6, 7, 8, 10, and 11 (H a), and H1, 2, 9, 12, 13, 14, and 15 (H b). The sample assignments to the groups a and b, respectively, are random. Blue numbers indicate Pearson correlation values. The averaged profiles show high correlations within the groups, and low correlations between the groups. **c** Principal component analysis (PCA) separates UC strongly from healthy controls in component 1 (39.9%)

The proteomes of UC and healthy tissue differed strongly, as shown in Fig. 1. We present the 20 proteins with the highest abundance increases and decreases, respectively, in Table 2. The Pearson correlations of normalized intensities (see Additional file 1) were higher when samples of the same group were compared (i.e. UC to UC, or healthy control to healthy control) than when UC samples were compared to healthy controls (Fig. 1a). Also the hierarchical clustering of the sample correlations resulted in separate grouping of UC and healthy samples (Fig. 1a). In addition averaged profiles showed high Pearson correlations (> 0.7) for comparisons within the groups UC and healthy, respectively, and low correlations (< 0.1) for comparisons of UC to healthy (Fig. 1b). Several multi scatter plots were investigated with random samples from UC patients or healthy controls in each group, and all showed the same trend with high correlations internally in the UC and healthy sample groups. Upon unsupervised investigation by principal component analysis (PCA) (Fig. 1c) a strong separation of UC and healthy in component 1 which explains 39.9% of the variance was observed. To investigate possible biases we controlled for grouping by gender, and such grouping did not occur.

**Table 2:** 20 proteins with highest abundance increase and decrease, respectively, in UC patients as compared to healthy controls

Ratio UC/H	Protein name	Gene names	Majority protein ID	− Log p value
*Proteins of increased abundance in UC compared to healthy controls*				
10.12	Kinesin-like protein	KIF26B	B7WPD9	10.55
6.72	Protein S100-A8	S100A8	P05109	8.41
6.70	Cathelicidin antimicrobial peptide	CAMP	J3KNB4	9.71
6.17	Protein S100-A9	S100A9	P06702	8.07
4.95	Protein S100-A12	S100A12	P80511	8.36
4.73	Neutrophil defensin 1	DEFA1	P59665	7.02
4.47	Lactotransferrin	LTF;HEL110	E7EQB2	9.42
4.39	Myeloblastin	PRTN3	P24158	7.49
4.18	Neutrophil gelatinase-associated lipocalin	NGAL;LCN2	B2ZDQ1	13.57
3.54	Neutrophil elastase	ELANE;ELA2	P08246	7.48
3.51	Azurocidin	AZU1	P20160	8.36
3.49	Cysteine-rich secretory protein 3	CRISP3	J3KPA1	6.44
3.22	Myeloperoxidase	MPO	P05164-2	9.05
3.20	Bactericidal permeability-increasing protein	BPI	A2NX48	4.31
3.18	60S ribosomal protein L14	RPL14	P50914	1.62
3.17	Bactericidal permeability-increasing protein	BPI	P17213	8.26
3.16	Lysozyme	LYZ	B2R4C5	8.50
3.12	Marginal zone B- and B1-cell-specific protein	MZB1	Q8WU39	14.01
3.07	Ig heavy chain V-I region EU	IGHV1-69-2	P01742	3.98
3.01	Ficolin-1	FCN1	O00602	6.65
*Proteins of decreased abundance in UC compared to healthy controls*				
0.12	Hydroxymethylglutaryl-CoA synthase, mitochondrial	HMGCS2	A0A140VJL2	14.77
0.17	Fatty acid-binding protein, liver	FABP1	Q6FGL7	14.41
0.19	3-keto-steroid reductase	HSD17B7;hCG_2024989	P56937-2	14.32
0.24	Sulfate transporter	SLC26A2	P50443	18.52
0.26	Tumor necrosis factor alpha-induced protein 8-like protein 3	TNFAIP8L3	Q5GJ75	5.42
0.29	Carbonic anhydrase 2	HEL-76;CA2	V9HW21	12.09
0.30	Selenium-binding protein 1	HEL-S-134P;SELENBP1	V9HWG1	15.36
0.33	cAMP-dependent protein kinase inhibitor beta	PKIB	Q5T0Z6	7.57
0.34	Chloride anion exchanger	SLC26A3;DKFZp686P10213	P40879	10.33
0.35	UDP-glucuronosyltransferase 2A3	UGT2A3	Q6UWM9	14.95
0.36	Chymotrypsin-C	CTRC	Q99895	5.37
0.36	Hyaluronan and proteoglycan link protein 3	HAPLN3	A0A024RC58	8.55
0.37	Calcium-activated chloride channel regulator 1	CLCA1	A8K7I4	6.68
0.37	Tissue alpha-l-fucosidase	FUCA1	P04066	10.42
0.37	Phospholipase A(2)	PLA2G1B	F8W062	2.71
0.38	UDP-glucuronosyltransferase 2B17	UGT2B17;UGT2B15	O75795	3.10
0.38	Aquaporin-8	AQP8	Q53GF6	7.86
0.38	Mimecan	OGN	Q7Z532	9.66
0.39	Cadherin-17	CDH17	Q12864	17.16
0.39	Alcohol dehydrogenase 1C	ADH1C	P00326	10.05

Included are only significantly different proteins as determined in the two-sample test comparing the groups UC patients and healthy controls (see Additional file 1). Only the first majority protein ID for each identification is given, for further IDs see Additional file 2

### Comparison with previous proteomics studies on UC

The in total 8562 identified proteins provided the so far deepest view into the proteome of human colon mucosa and of UC. We report the abundance changes including p values for all quantified proteins (see Additional file 2: Excel file). 6818 of these proteins were quantified in at least 70% of the samples and were subject to enrichment analysis. Our data confirmed and extends previous findings, but also includes novel findings, such as the increased abundance of signal peptidase complex proteins. We searched PubMed for the terms “ulcerative colitis proteomics” and reviewed the titles and abstracts to select the relevant studies. Additionally we included studies previously known to the authors, totaling to 7 studies for the comparison [3–9]. The present study was largely in agreement with those previous studies about the direction of abundance changes of differently abundant proteins. However, the magnitudes of the changes differed.

### Functional analysis

**Fig. 2:**
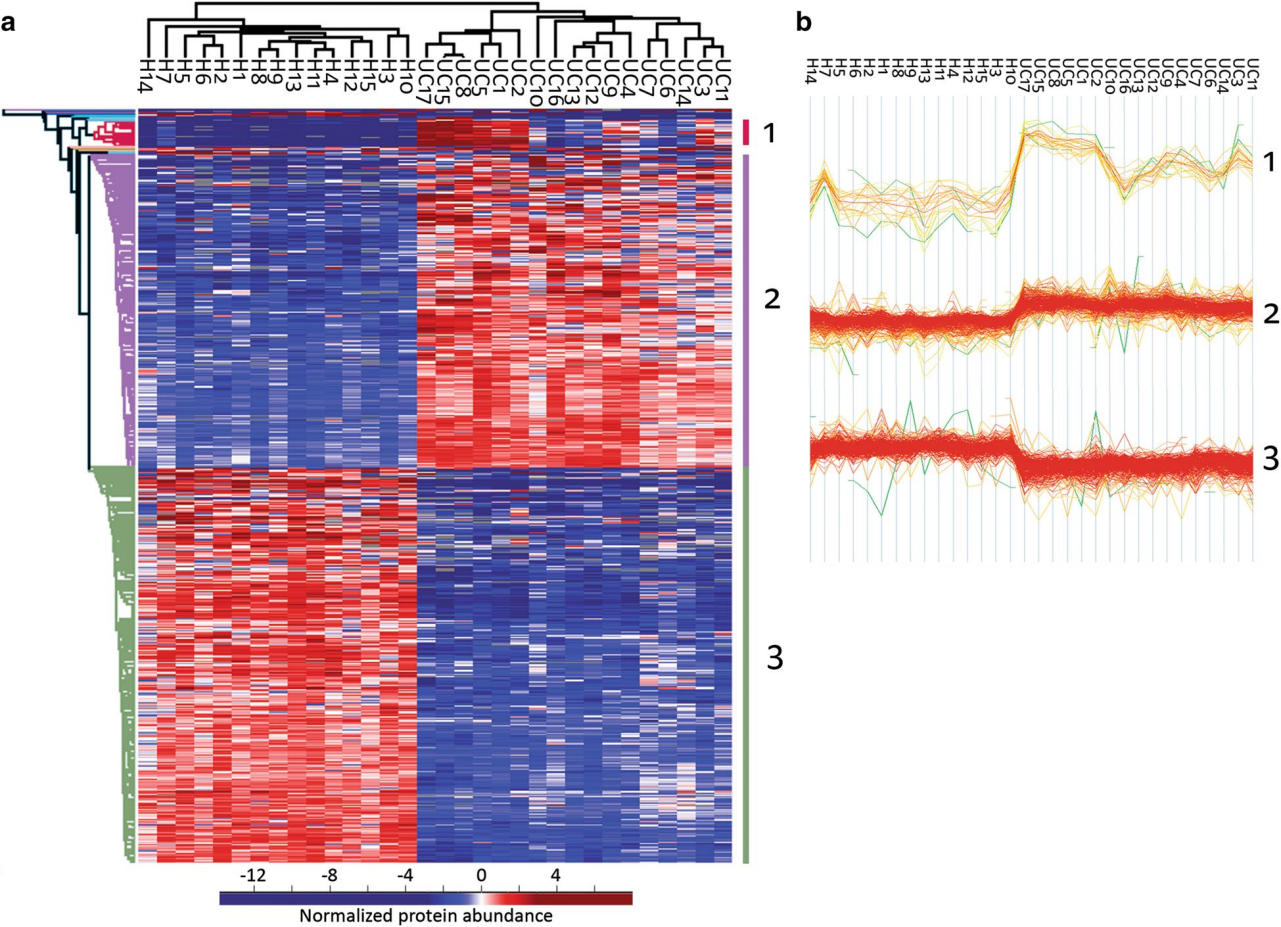
Clustering of the 596 most differently abundant proteins between ulcerative colitis (UC) and healthy controls. **a** Hierarchical clustering reveals three main protein clusters. Low abundances are indicated in blue, high abundances in red. **b** Protein abundance profiles of the three main clusters. 1: minor cluster of 20 proteins (including calprotectin) with increased abundance in UC, with a subgroup of UC samples showing a more pronounced increase. 2: major cluster of 247 proteins with increased abundance in UC. 3: Cluster of 313 proteins with a decreased abundance in UC

Hierarchical clustering of the 596 most differently abundant proteins (8.7% of all proteins quantified in > 70% of samples; s0 = 2, FDR = 0.01) revealed three major clusters (Fig. 2). The proteins which were higher abundant in UC divided into two clusters, a small cluster of 20 proteins with a more pronounced abundance change in a subgroup of samples, and a large cluster of 247 proteins. The lower abundant proteins formed one cluster of 313 proteins. In total 16 proteins were outside these three clusters.

The extraction of biological information from omics datasets is a major challenge. Enrichment analyses can overcome some of the difficulties [10]. Enrichment analyses annotate categorical terms (e.g. functions, localizations, pathways) to proteins and subsequently determine which terms are overrepresented among the differently abundant proteins compared to the entire dataset of quantified proteins. Knowledge about the involved pathways and functions can help to understand the disease, understand complications, and develop new treatment options.

Abundance changes found in tissue proteomics could result from differential regulation of the protein abundances in cells, but also from changes of the cell population (e.g. from the migration of immune cells into inflamed tissue in UC [11]) or the extracellular matrix. It seems plausible that each of these factors affects all measured abundances in such an experiment to varying degrees.

Lists of the enriched gene ontology terms among proteins which were found to be differently abundant in UC as compared to healthy tissue, and a list of the proteins which contributed to the functions discussed in the following are provided as Additional file 2: Excel file.

This article can neither cover all enriched terms we found, nor can it discuss the impact of each differently abundant protein on a function or the magnitude of its abundance change in detail. We therefore strongly encourage the reader to consult the above mentioned tables for further information on functions and proteins of interest.

### Lower abundant in UC

**Fig. 3:**
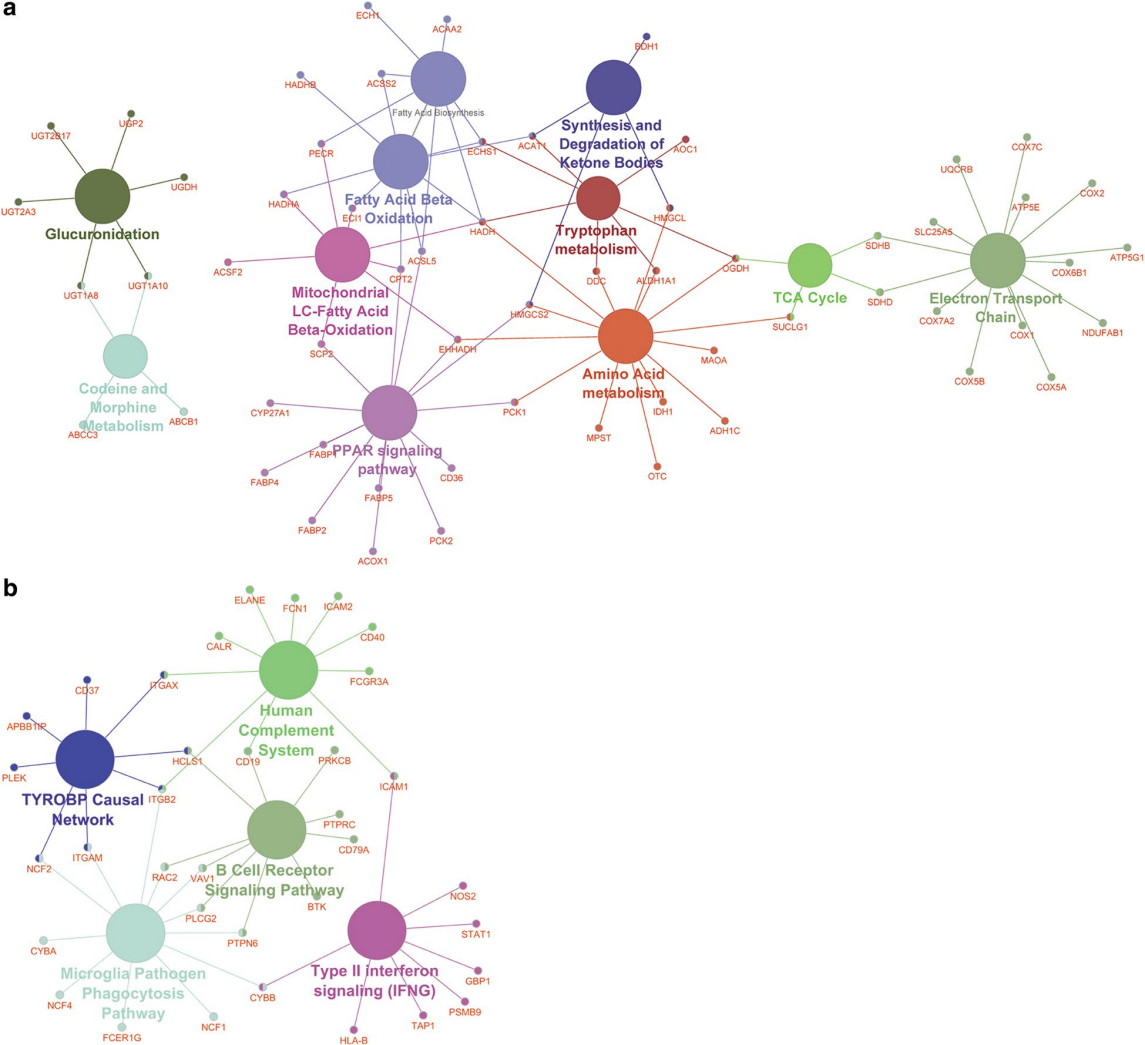
Functional networks of differently abundant proteins in colon mucosa biopsies affected by ulcerative colitis compared to healthy controls. **a** Functional network of the 321 proteins with the strongest abundance decrease in ulcerative colitis compared to healthy tissue. Metabolic functions dominate this network, especially those located in the mitochondria. **b** Functional network of the 275 proteins with highest abundance increase in ulcerative colitis. Functions of the immune system characterize this network. Generated with ClueGO, Pathways: WikiPathways (updated: 10.03.2018), pV ≤ 0.01

321 proteins showed decreased abundances in UC as compared to healthy control samples. 313 of these grouped into one cluster (Fig. 2, cluster 3). Functional analysis revealed that metabolic pathways were overrepresented among the proteins with decreased abundance in UC. The enrichment of proteins with decreased abundances in our dataset affects short-chain and long-chain fatty acid, ketone body and amino acid (particularly tryptophan) metabolism as well as the tricarboxylic acid (TCA) cycle (see Additional file 1: SuppPathway1–4) and the electron transport chain (ETC; Fig. 3a).

The observed abundance decreases of butyrate utilizing proteins are in line with previous studies which show a decreased butyrate metabolism by UC tissue. Butyrate is a short-chain fatty acid (SCFA) and the main energy source of healthy colon mucosa [31]. It was previously hypothesized that ulcerative colitis is an energy-deficiency disease resulting from the failure to utilize butyrate [31]. Colonocytes convert a part of the butyrate to ketone bodies [32]. Our data showed that proteins related to this specific utilization of butyrate were lower abundant in UC. Several studies have investigated whether butyrate has a positive effect on inflamed colon tissue, however the results of these studies are conflicting [33].

The decreased protein abundances also affected the transport and metabolism of the long-chain fatty acids (LCFA), i.e. the long carbon chain analogs of SCFAs. LCFAs have an ambiguous role in ulcerative colitis. Linoleic acid increases the chances of developing ulcerative colitis [34]. Omega-3 fatty acids, such as docosahexaenoic acid (DHA), may reduce the chance of developing the disease [35]. Studies on the efficacy of omega-3 fatty acids in the treatment of ulcerative colitis show controversial results. Systematic reviews however conclude that large high-quality studies fail to show positive effects, and that there is no sufficient evidence for the efficiacy of omega-3 fatty acids in the treatment of UC [36, 37].

Mitochondrial enzymes involved in the metabolism of SCFA and LCFA as well as the tricarboxylic acid (TCA) and the electron transport chain (ETC) were found in our dataset to be low abundant in UC. These findings reflect a mitochondrial dysfunction which possibly contributes to the proposed energy deficiency [38].

Proteins related to nuclear receptors were of decreased abundance in UC. Peroxisome proliferator-activated receptor (PPAR)-inducible proteins were lower abundant in UC (Fig. 3). The changes in the LCFA transport and the PPAR pathway are interdependent. Activation of the intracellular PPAR receptors by LCFA demands the import of the fatty acids into the cell and the transport within the cell. One protein involved in the transport into the cell is CD36 which is low abundant in UC tissue. Fatty acid binding proteins (FABP) facilitate the transport within the cell [39]. Four FABPs (FABP1, FABP2, FABP4, and FABP5) are among the proteins with the lowest relative abundance in UC tissue compared to healthy tissue. A decreased transport of LCFA might in turn be partially causative for the decreased abundances of PPAR-inducible proteins, such as ACOX1, HMGCS2, FABP1, and PCK1 [40]. In accordance with a previous study [41], we found the vitamin D3 receptor NR1i1 to be lower abundant in UC tissue. However, we observed no general abundance changes of proteins regulated by this receptor. Previous studies found decreased abundances of hepatocyte nuclear factor 4 alpha (HNF4A) in UC and suggested a role in the disease pathophysiology [42]. Our data supports these findings.

All detected metallothioneins—MT1H, MT2A, and MT1F—were found to be lower abundant in UC tissue compared to healthy controls. Metallothioneins are small, cysteine-rich proteins, which bind heavy metal cations. Our findings confirmed several previous studies, which concluded that the decreased metallothionein abundances may result in an inefficient antioxidant response in the mucosa and in turn contributes to the IBD pathophysiology [43]. However, other studies present conflicting results and conclusions [44].

Histamine is the major mediator of mast cells and contributes to the immuno-inflammatory reaction in IBD. Previous studies showed increased histamine secretion and levels in UC affected tissues [45] and decreased histaminase (AOC1) activity [9]. The present study revealed that both major histamine degrading enzymes—Histaminase (AOC1) and histamine *N*-methyltransferase (HNMT)—were lower abundant in UC tissue compared to healthy tissue (not based on enrichment analysis). This finding constitutes a plausible causative explanation for some of the previously reported findings on histamine in UC.

Chemical compounds undergo phase I and phase II biotransformation reactions which make them more hydrophilic [46]. This allows their excretion with urine because the polar compounds are not reabsorbed in the kidneys [47]. Phase I reactions introduce or change functional groups by oxidation, reduction, or hydrolysis. In phase II reactions, enzymes attach hydrophilic endogenous compounds [46]. Our data showed that the abundances of a large proportion of both phase I and phase II enzymes are decreased in UC. A decrease of xenobiotic metabolism gene products in UC was shown earlier on mRNA level [48]. The affected phase II enzymes are involved in sulfonation, glucuronidation, and glutathione conjugation. Proteins related to both phase 1 (monoamine oxidase A, MAOA) and phase 2 metabolism (sulfotransferases) of catecholamines were found in our dataset to be less abundant in UC. This could contribute to previously found increases of norepinephrine in rectal UC mucosa [49].

Synthetic steroids are an important treatment option for ulcerative colitis. Our data showed impairments in the steroid metabolism in UC. This suggests a role of endogenous steroids in the pathophysiology of the disease. The hydroxysteroid dehydrogenases HSD11B2, HSD17B11, HSD17B2, and HSD17B7 were among the most down-regulated proteins in UC (HSD17B8 is downregulated to a lesser degree). The abundances of HSD17B4, HSD17B10, and HSD17B12 were not different in UC from healthy tissue. Decreased abundances of HSD11B2 [50] and HSD17B7 [3] have been reported previously. Our data furthermore showed that proteins related to the transport of bile acids are enriched among the lower abundant proteins, which supports findings of a previous study [41]. Pathway mapping shows abundance decreases of proteins involved in the neutral pathway of bile acids biosynthesis (Additional file 1: SuppPathway5). However, the implications of this finding are not clear, because bile acids are primarily synthesized in hepatocytes, whereas there is no evidence of bile acid synthesis in enterocytes [51].

The carbonic anhydrases CA1, CA2, CA4, and CA12 were found to be lower abundant in UC. CA3 was the only quantified but not significantly lower abundant carbonic anhydrase. A decreased abundance in UC was reported earlier for CA1 [52].

Fibrillar collagens are a subgroup of collagens and provide three-dimensional frameworks for tissues and organs [53]. The fibrillar collagens were enriched among the proteins that were of decreased abundance in UC. Proteins that are involved in collagen degradation, e.g. metalloproteinases, on the other hand were enriched among the proteins with increased abundance in UC (see below). This finding is in accordance with a previous study on the role of collagen degradation in IBD [54].

### Higher abundant proteins in UC

As expected, many proteins with increased abundance in UC are involved in inflammatory and immune processes (Fig. 3b). Proteins related to neutrophils (e.g. NADPH oxidase complex proteins which generate superoxide, metalloproteinases) and B cells (e.g. V and C regions of immunoglobulins) were highly enriched. This could result from a tissue infiltration by these immune cells.

The higher abundant proteins divided into two main clusters under hierarchical clustering (Fig. 2, clusters 1 and 2). The UC patients with high cluster 1 protein abundances (UC1, UC2, UC5, UC8, UC15, UC17) grouped together (Fig. 2a, b). Cluster 1 contained proteins occurring in neutrophils, among these Protein S100A8 and Protein S100A9 which together form the calprotectin complex (see Additional file 1: Table 3). Several proteins (calprotectin, S100A12, lactotransferrin) of the minor cluster have been used to differentiate between IBD and non-IBD [55]. Interestingly, the average Geboes index in the patient cluster with high cluster 1 protein abundances is 11.0 (SD = 3.1), while it is 6.5 (SD = 3.2) for the other patients.

Our data showed that inducible nitric oxide synthase (NOS2) is higher abundant in UC tissue (not based on enrichment analysis). NOS2 produces nitric oxide (NO) which has various physiological roles as a messenger molecule. It functions most prominently as a vasodilator [56], but it is also involved in the immune system [57]. NO is directly toxic to pathogens, induces or suppresses apoptosis, and regulates the immune reaction [58]. Excess NO is cytotoxic and induces cell death. NOS2 is inducible by inflammation, infection and other stimuli. Increased NOS2 abundances in UC were shown earlier by immunostaining [8] and our results confirm these findings.

Proteins with functions in protein processing in the endoplasmic reticulum (ER) are overrepresented among the proteins which were more abundant in UC. Also unfolded protein response (UPR) and signal peptidase complex (SPC) proteins are related to protein processing, located in the ER, and enriched among proteins with increased abundances in UC. UPR has previously been identified as one factor in the pathophysiology of ulcerative colitis [59]. The enrichment of SPC proteins among proteins with increased abundances in UC is a novel finding from our data.

However, ER proteins were not generally higher abundant. For instance, many metabolic enzymes are located in the ER and enriched among the proteins with decreased abundances (e.g. UDP-glucuronosyltransferases, see “Lower abundant in UC” section).

The SPC is not inherently inflammatory. Nonetheless its proteins were among those with the highest abundance increases in UC. The SPC is located in the membrane of the endoplasmic reticulum and cleaves the signal peptide cotranslationally from nascent proteins [60]. The SPC subunits SPCS1, SPCS2, and SPCS3 and the SPC catalytic subunit SEC11C were more abundant in UC. They furthermore showed highly similar abundance profiles (Additional file 1: Figure 3). The SPC catalytic subunit SEC11 however does not follow this pattern and was similarly abundant in UC and healthy tissue.

Accumulation of unfolded and misfolded proteins causes stress to the endoplasmic reticulum. The UPR aims to remove these proteins. To achieve this, the cell stops the translation of further proteins, degrades misfolded proteins, and produces chaperones, which correct the protein folding. Our data showed strong enrichment of proteins related to UPR, response to ER stress, and the ER chaperone complex among the higher abundant proteins in UC. Specifically, several chaperones that can be induced by ATF6 alpha (HSP90B1, calreticulin [61], and HSPA5 [62]), which plays a central role in UPR, were enriched. Our findings support previous assumptions that the UPR may play a role in the pathophysiology of UC.

### Foreseeable applications

The functional changes we observed in the present study allow for hypothesis generation for treatment approaches.

Alterations of metabolic pathways in ulcerative colitis imply that nutritional interventions directed at the respective metabolites could be effective in the treatment of the disease. For instance, decreased metallothionein abundances indicate that the detoxification of heavy metals and the utilization of polyvalent metal ions could be impaired. Considering the decreased detoxification capacities, reduced intake or chelation of toxic heavy metals might have beneficial effects. On the other hand, the supplementation of essential polyvalent metal ions could be advantageous. Furthermore, the decreased metabolic and transport capabilities related to butyrate contribute probably to the energy deprivation of colonocytes. Previous studies investigated the treatment of UC with butyrate and other SCFA. But the pathophysiological changes we observed related to the utilization of those compounds imply that supplementation with a later metabolite such as beta-hydroxybutyrate might be preferable. Beta-hydroxybutyrate is a ketone body that becomes systemically available after oral administration and has been used in the treatment of other pathophysiological conditions [63, 64].

The enrichment of proteins related to signaling pathways such as PPAR designates the respective pathways as potential drug targets.

Abundance changes of single proteins may have similar implications, especially if they are known to be drugable. Proteins with high abundance differences between UC patients and healthy controls and high corresponding − Log p values are of potential interest as biomarker candidates. Proteins with these characteristics are for instance SLC26A2, HMGCS2, and CD38 (Additional file 1: Figure 4; Additional file 2).

However, further testing, clinical studies, and validation will be necessary before any clinical application.

## Conclusions

Our study presents new evidence and complements previous findings about changes of biological functions in UC. The major novel findings of this study were the increased abundances of SPC proteins and the presence of two distinguished clusters of higher abundant proteins, with the calprotectin complex proteins S100A8 and S100A9 in the minor cluster 1. This cluster of 20 proteins diverged from the abundance changes of the majority of proteins with increased abundances in UC that we observed in cluster 2.

In conclusion, abundances in UC tissue were increased compared to healthy controls for proteins related to the immune system and to protein processing in the ER, e.g. UPR and SPC proteins. Immune cell infiltration into the inflamed tissue contributes probably to the increased immune system protein abundances. The high abundance of UPR proteins indicates an ER stress response. Moreover, NOS2 which produces NO was higher abundant in UC tissue.

Lower abundant in UC were predominantly metabolic proteins. These metabolic proteins comprise mitochondrial enzymes for the metabolization of SCFA and LCFA as well as proteins of the TCA cycle and the ETC. These changes are probably contributing to the previously reported mitochondrial dysfunction and energy deficiency of colonocytes in UC. The abundances of LCFA transport proteins were decreased which may be partially causative for the decreased abundances of PPAR-inducible proteins. Decreased abundances of histamine degrading enzymes probably contribute to the increased histamine levels in UC tissue which were found in previous studies. Furthermore, abundances of proteins involved in phase I and phase II biotransformation were decreased, as well as the abundances of several metallothioneins, hydroxysteroid dehydrogenases, and carbonic anhydrases.

We provide a list of all 8562 identified proteins including the fold changes UC/H and p values as supporting information (see Additional file 2: Excel file).

## Additional files

**Additional file 1.** Detailed method descriptions, additional figures and tables, mapping of metabolic pathways.

**Additional file 2.** Complete list of identified proteins including UC/Healthy ratios and p-values, lists of gene ontology term enrichments, enriched functions discussed in the article and their proteins.

## Abbreviations

BCA: bicinchoninic acid
DHA: docosahexaenoic acid
ER: endoplasmic reticulum
ETC: electron transport chain
FA: formic acid
FABP: fatty acid binding protein
IBD: inflammatory bowel disease
LCFA: long-chain fatty acid
Lys-C: endoproteinase Lys-C
NO: nitric oxide
PCA: principal component analysis
PPAR: peroxisome proliferator-activated receptor
SCFA: short-chain fatty acid
SDC: sodium deoxycholate
SPC: signal peptidase complex
TCA cycle: tricarboxylic acid cycle
TEAB: triethylammonium bicarbonate buffer
TMT: tandem mass tag
UC: ulcerative colitis
UPR: unfolded protein response
