# Supplementary material for: Ulcerative colitis: functional analysis of the in-depth proteome
Source: Clin Proteomics. 2019 Jan 29;16:4. doi: 10.1186/s12014-019-9224-6 (PMC6350310; doi:10.1186/s12014-019-9224-6)
Supplement: Supplementary file 1 — Additional file 1. Detailed method descriptions, additional figures and tables, mapping of metabolic pathways. [file 12014_2019_9224_MOESM1_ESM.docx]

# Supplementary Data Content 1

# Supplementary texts

# LC-MS/MS

## Materials

LC-MS/MS was performed with an EASY-nLC 1000 coupled to a Q Exactive (both Thermo Fisher Scientific, Bremen, Germany).

The LC was equipped with an Acclaim PepMap® 100 precolumn, C18, particle size 3 μm, pore size 100 Å, inner diameter 75 μm, length 2 cm, nanoViper,(Thermo Fisher Scientific, Bremen, Germany). The separation column was an EASY-Spray, PepMAP® RSLC, C18, particle size 2 μm, pore size 100 Å, inner diameter 75 μm, length 50 cm (Thermo Fisher Scientific, Bremen, Germany).

The solvents for separation were water with 0.1% FA, and acetonitrile with 0.1% FA, respectively.

## LC settings

The peptides were concentrated on a reversed-phase trap column with 0.1% formic acid (FA) at a flow rate of 20 µl/min. The peptides were separated on a reversed-phase main column with a binary solvent gradient. The column temperature was set to 60 °C and the flow rate to 200 nl/min. The ACN proportion was increased from 2% to 5% over 19 min, further to 30% at 180 min and to 100% at 200 min. The column was regenerated with 100% ACN for additional 10 min.

## MS/MS settings

The Q Exactive mass spectrometer was run in positive mode with the following settings: Chromatographic peak width 15 s, default charge state 2, full MS survey scans from 400 to 2,000 m/z, resolution 70,000, AGC target value 3e6, maximum injection time 100 ms for MS scans. Subject the 10 most intense peaks to MS/MS with the following settings: resolution 17,500, dynamic exclusion 10 s, underfill ratio 1%, charge states +2, +3 and +4, exclude isotopes, normalized collision energy 31, isolation window 2 m/z, AGC target value 1e5 and maximum injection time 50 ms, fixed first mass 120.

# MaxQuant settings

The mass spectrometry data was searched in MaxQuant version 1.6.1.0 against a fasta file from <https://www.uniprot.org/> (download date: 24.11.2017). This file included all human proteins (canonical and isoforms). The TMT reporter ion distribution was configured according to the manufacturer’s Certificate of Analysis. The quantification method was set to “Reporter ion MS2/TMTsixplex”. File names and fraction numbers were set according to the experimental setting. The following further parameters were applied:

Enzyme: Trypsin/P (specific), fixed modifications: Carbamidomethyl (C), variable modifications: Oxidation (M) and Acetyl (Protein N-term), max. 2 missed cleavages, PSM FDR: 0.01, Protein FDR: 0.01 Site FDR: 0.01, Use Normalized Ratios For Occupancy: TRUE, Min. peptide Length: 7, Min. score for unmodified peptides: 0, Min. score for modified peptides: 40, Min. delta score for unmodified peptides: 0, Min. delta score for modified peptides: 6, Min. unique peptides: 0, Min. razor peptides: 1, Min. peptides: 1, Use only unmodified peptides and: TRUE, Modifications included in protein quantification: Oxidation (M) and Acetyl (Protein N-term), Peptides used for protein quantification: Razor, Discard unmodified counterpart peptides: TRUE, Label min. ratio count: 2, Use delta score: FALSE, iBAQ: FALSE, iBAQ log fit: FALSE, Match between runs: TRUE, Matching time window [min]: 0.7, Alignment time window [min]: 20, Find dependent peptides: FALSE, Decoy mode: revert, Include contaminants: TRUE, Advanced ratios: TRUE, Second peptides: FALSE, Calculate peak properties: FALSE, Main search max. combinations: 200, Advanced site intensities: TRUE, Max. peptide mass [Da]: 4600, Min. peptide length for unspecific search: 8, Max. peptide length for unspecific search: 25, Razor protein FDR: TRUE, Disable MD5: FALSE, Max mods in site table: 3, Match unidentified features: FALSE, MS/MS tol. (FTMS): 20 ppm, Top MS/MS peaks per Da interval. (FTMS): 12, Da interval. (FTMS): 100, MS/MS deisotoping (FTMS): TRUE, MS/MS deisotoping tolerance (FTMS): 7, MS/MS deisotoping tolerance unit (FTMS): ppm, MS/MS higher charges (FTMS): TRUE, MS/MS water loss (FTMS): TRUE, MS/MS ammonia loss (FTMS): TRUE, MS/MS dependent losses (FTMS): TRUE, MS/MS recalibration (FTMS): FALSE.

# Perseus

## Data normalization

The proteinGroups file generated by MaxQuant was loaded into Perseus version 1.6.1.1. The “Reporter Intensity Corrected” columns were loaded as main columns. These intensities were log(2) transformed. The respective standard intensities were then subtracted. The intensity columns were renamed according to the respective sample name and categorical annotations were added (UC and H, respectively). Rows were filtered to remove proteins labelled as “Only identified by site”, “Reverse” and “Potential contaminant”. The intensities were then Z-score normalized (matrix access: column).

The Z-score normalized values were used for all analyses, except the determination of the UC/H ratio.

## Creation of lists and figures

### Figures

For figure 1A the rows were filtered to have at least 70% values and column correlations were calculated for the normalized protein intensities with standard settings. The results were visualized by hierarchical clustering. The replicates were only included for this figure and were removed for all other analyses.

For figure 1B, the Z-score transformed dataset was filtered for 70% valid values. The intensities of each protein for UC2, 3, 4, 6, 7, 11, 13, 14, and 16 (UC a), UC1, 5, 8, 9, 10, 12, 15, and 17 (UC b), H3, 4, 5, 6, 7, 8, 10, and 11 (H a), and H1, 2, 9, 12, 13, 14, and 15 (H b), respectively, were averaged. This was done by assigning the respective groups to the samples, followed by the function “Annot. rows → Average groups, Average type: mean”. The averaged intensities were visualized in a multi scatter plot.

For figure 1C, the dataset without replicates was filtered for 100% valid values before the PCA was generated.

Figure 2 was generated by filtering the normalized dataset without replicates for 70% valid values. A two-sample test was performed to determine significant differences between the UC samples and healthy controls with standard settings, except for s0 = 2 and FDR = 0.01. The dataset was then filtered for significantly changed proteins. The figure was prepared by hierarchical clustering of the resulting dataset, and setting the number of clusters to 17.

For figure 3A and B, the lower abundant proteins and higher abundant proteins, respectively, which were identified in the previous two-sample test were selected. Their gene identifiers were loaded into the Cytoscape app ClueGO and analyzed against WikiPathways (updated 07.02.2018), showing only pathways with pV ≤ 0.01. GO term grouping was activated for the higher abundant proteins, but not for the lower abundant proteins. The ClueGO Layout was selected and manually refined. All other parameters were at the standard setting.

Metabolic pathways (suppPathway1-5) were mapped at [https://humancyc.org/overviewsWeb/celOv.shtml#](https://humancyc.org/overviewsWeb/celOv.shtml) (date: 01.01.2019) with Gene IDs and logaritmized UC/H ratios of all quantified proteins.

### List of enriched GO terms

To generate the enrichment lists provided with the supporting information, annotations were added to the unfiltered matrix resulting from the two sample test (see preparation of Figure 2). Lists of more and less, respectively, abundant proteins were generated, based on the two-sample test. These lists were separately matched back to the matrix with the added annotations with the function “matching rows by name” and the option of an indicator in the new matrix. Fisher exact tests with the categorical column indicating the respective matrix of enriched proteins and standard settings gave the enrichment lists.

### List of proteins in enriched terms

For the ratio UC/H, the normalized intensities **before Z-scoring** (i.e. after log(2) transformation and subtraction of the respective standard intensity) were used. The mean of the sample intensities (matrix acces: column) was subtracted. The difference between the normalized log(2) intensities of UC and H was calculated in a two-sample test. The resulting log(2) differences were transformed back by a 2^x operation which gives the ratio of the normalized protein intensities.

The UC/H ratio column was then matched with the function “Matching rows by name” to the column “majority protein” into a matrix of the 6818 proteins which were quantified in >70% of the samples, including two-sample test results (after Z-scoring) and GO annotations.

A volcano plot was created from this list. The rider “categories” allows the selection of all proteins in a given category by clicking on the category name in the list. The respective proteins were subsequently exported into a separate matrix (Rider: “Points” → “Export selection (reduce matrix”), which was then exported into a tab delimited text file (Right-click → “Plain matrix export”).

### List of all proteins

The Z-score difference and the –log p-value were determined in a two-sample test from the normalized values after Z-scoring from all 8562 identified proteins. The corresponding Ratio UC/H was calculated and implemented as described in “List of proteins in enriched terms”. The indicators for whether a difference is significant (as generated for figure 2) were matched into this matrix with the function “Matching rows by name/Matching column: Majority Proteins IDs”.

# Supplementary tables

| **Grade** | **Average** | **SD** | **Range** |
| --- | --- | --- | --- |
| Architectural changes (0-3) | 1.06 | 0.66 | 0-3 |
| Chronic inflammatory infiltrate (0-3) | 1.31 | 0.46 | 1-2 |
| Eosinophils in lamina propria (0-3) | 0.88 | 0.60 | 0-2 |
| Neutrophils in lamina propria (0-3) | 1.06 | 0.83 | 0-2 |
| Neutrophils in epithelium  (0-3) | 1.69 | 0.68 | 1-3 |
| Crypt destruction (0-3) | 1.31 | 1.04 | 0-3 |
| Erosions and ulcerations (0-4) | 0.63 | 1.36 | 0-4 |
| Total (0-22) | 7.94 | 3.77 | 3-16 |

Suppl. table1: Averages, standard deviations, and ranges of Geboes index grades for the UC patients.

| **Starting protein concentration [µg/ml] in sample as determined in BCA assay** | **TMTsixplex for 25 µg peptides** |
| --- | --- |
| ≥ 2200 | 0.4 mg |
| 1530 to 2200 | 0.5 mg |
| 1173 to 1530 | 0.6 mg |
| 952 to 1173 | 0.7 mg |
| 800 to 952 | 0.8 mg |

Suppl. table2: Used TMTsixplex amounts in dependence of starting concentration of samples. The TMTsixplex amount was adjusted for low concentrated samples, because poor labeling was observed otherwise during the method development.

| **Ratio UC/H** | **-log p-value UC_K** | **Protein names** | **Gene names** | **Majority protein IDs** |
| --- | --- | --- | --- | --- |
| 10.1177 | 10.5488 | Kinesin-like protein | KIF26B | B7WPD9 |
| 6.7208 | 8.40544 | Protein S100-A8 | S100A8 | P05109 |
| 6.69903 | 9.70578 | Cathelicidin antimicrobial peptide | CAMP | J3KNB4 |
| 6.16675 | 8.07071 | Protein S100-A9 | S100A9 | P06702 |
| 4.94808 | 8.35806 | Protein S100-A12;Calcitermin | S100A12 | P80511 |
| 4.72862 | 7.01854 | Neutrophil defensin 1 | DEFA1 | P59665 |
| 4.47297 | 9.41786 | Lactotransferrin | LTF;HEL110 | E7EQB2 |
| 4.39275 | 7.48579 | Myeloblastin | PRTN3 | P24158 |
| 4.17913 | 13.5737 | Neutrophil gelatinase-associated lipocalin | NGAL;LCN2 | B2ZDQ1 |
| 3.5434 | 7.48418 | Neutrophil elastase | ELANE;ELA2 | P08246 |
| 3.50704 | 8.35501 | Azurocidin | AZU1 | P20160 |
| 3.49049 | 6.43954 | Cysteine-rich secretory protein 3 | CRISP3 | J3KPA1 |
| 3.21954 | 9.05008 | Myeloperoxidase | MPO | P05164-2 |
| 3.2025 | 4.30977 |  | BPI | A2NX48 |
| 3.16036 | 8.49697 | Lysozyme | LYZ | B2R4C5 |
| 3.01386 | 6.64713 | Ficolin-1 | FCN1 | O00602 |
| 2.85388 | 6.53514 | Resistin | RETN | Q9HD89 |
| 2.72326 | 6.35354 |  |  | B7Z507 |
| 2.53399 | 6.33617 | Matrix metalloproteinase-9 | MMP9 | P14780 |
| 2.43248 | 5.56569 | Neutrophil collagenase | MMP8 | P22894 |

Suppl. table3: Proteins from the minor cluster of the proteins which are more abundant in UC (cluster 1, see article figure 2). Given for each protein is only the first Majority protein ID states (see Excel file, Supplementary Data Content 2, which lists all 8562 identified protein, for further IDs and data on these proteins (labeled there with “Cluster 1 (minor cluster increased abundance)“)).

# Supplementary figures


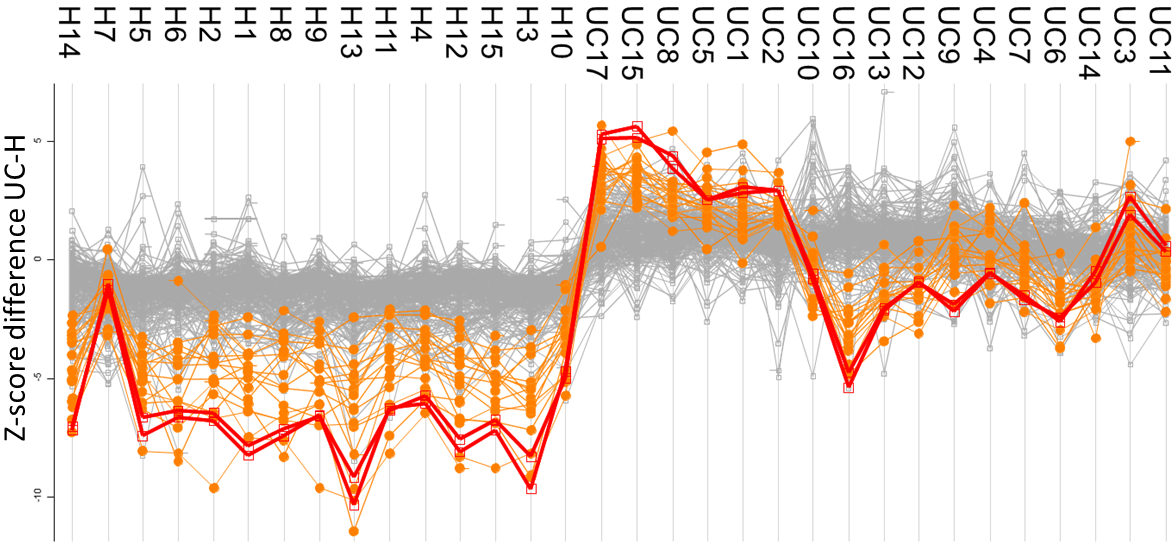


Suppl. figure1: Profile plot of the minor cluster (orange and red) of higher abundant proteins in UC in comparison with the major cluster of more abundant proteins (grey). The profile plots of S100A8 and S100A9, which form calprotectin, are shown in red.


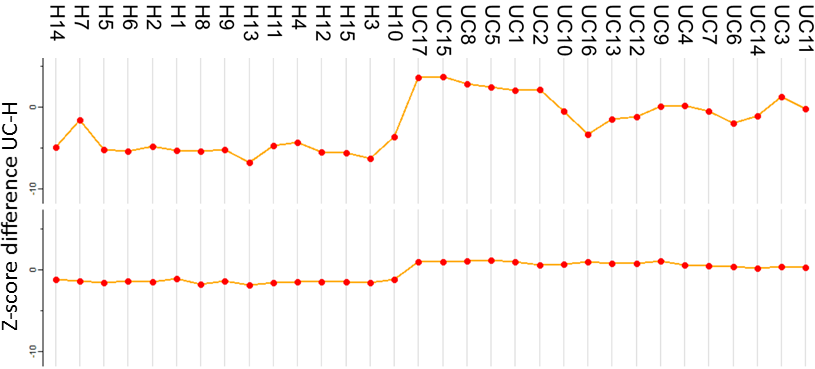


Suppl. figure2: Profile plot of the averages of the normalized protein intensities from the minor (top) and major (bottom) cluster of upregulated proteins in UC.


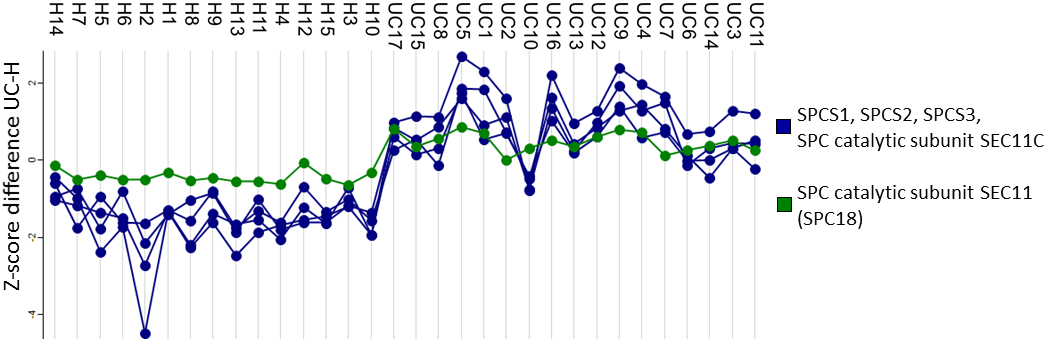


| Ratio UC/H | p-value UC_H | Gene name |
| --- | --- | --- |
| 1.63153 | 9.60672 | SPCS1 |
| 1.66381 | 11.2304 | SPCS3 |
| 1.70966 | 10.7076 | SPCS2 |
| 2.252 | 10.7787 | SEC11C |
| 1.26938 | 12.2656 | SPC18 |

Suppl. figure3: Profile plot and statistics of signal peptidase complex protein abundances. SPCS1, SPCS2, SPCS3, and SEC11C present similar abundance profiles. SPC18 does not follow this pattern and its abundance increase in UC compared to healthy tissue is lower.


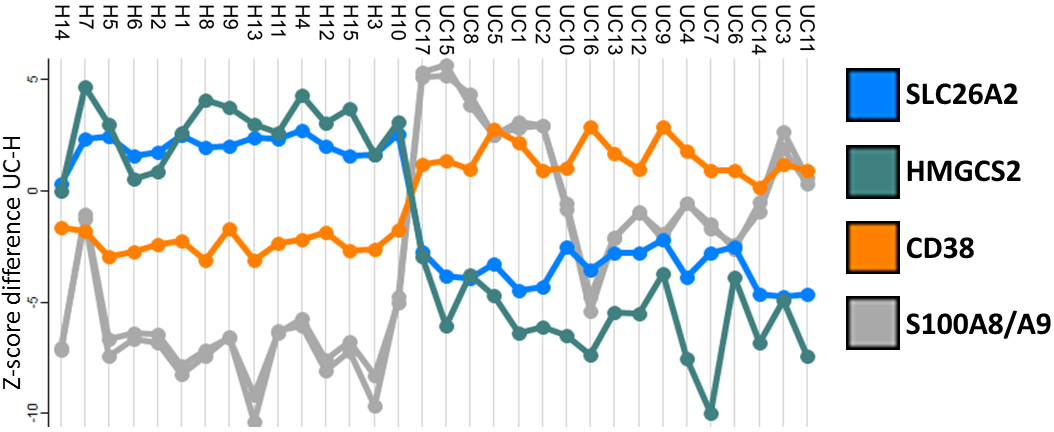


Suppl. figure4: Profile plot of normalized abundances of three proteins which differ strongly between UC and healthy tissue (SLC26A2, sulfate transporter; HMGCS2, Hydroxymethylglutaryl-CoA synthase; CD38, ADP-ribosyl cyclase/cyclic ADP-ribose hydrolase 1). These were selected based on p-value and the difference between the group averages. Calprotectin (S100A8 and S100A9) is shown for reference. It shows a large difference between the UC and healthy average intensities, but it presents with low significance. Overlaps occur in which single healthy tissue samples show higher calprotectin abundances than single UC samples (e.g. H7 and UC16).

# Mapping of metabolic pathways


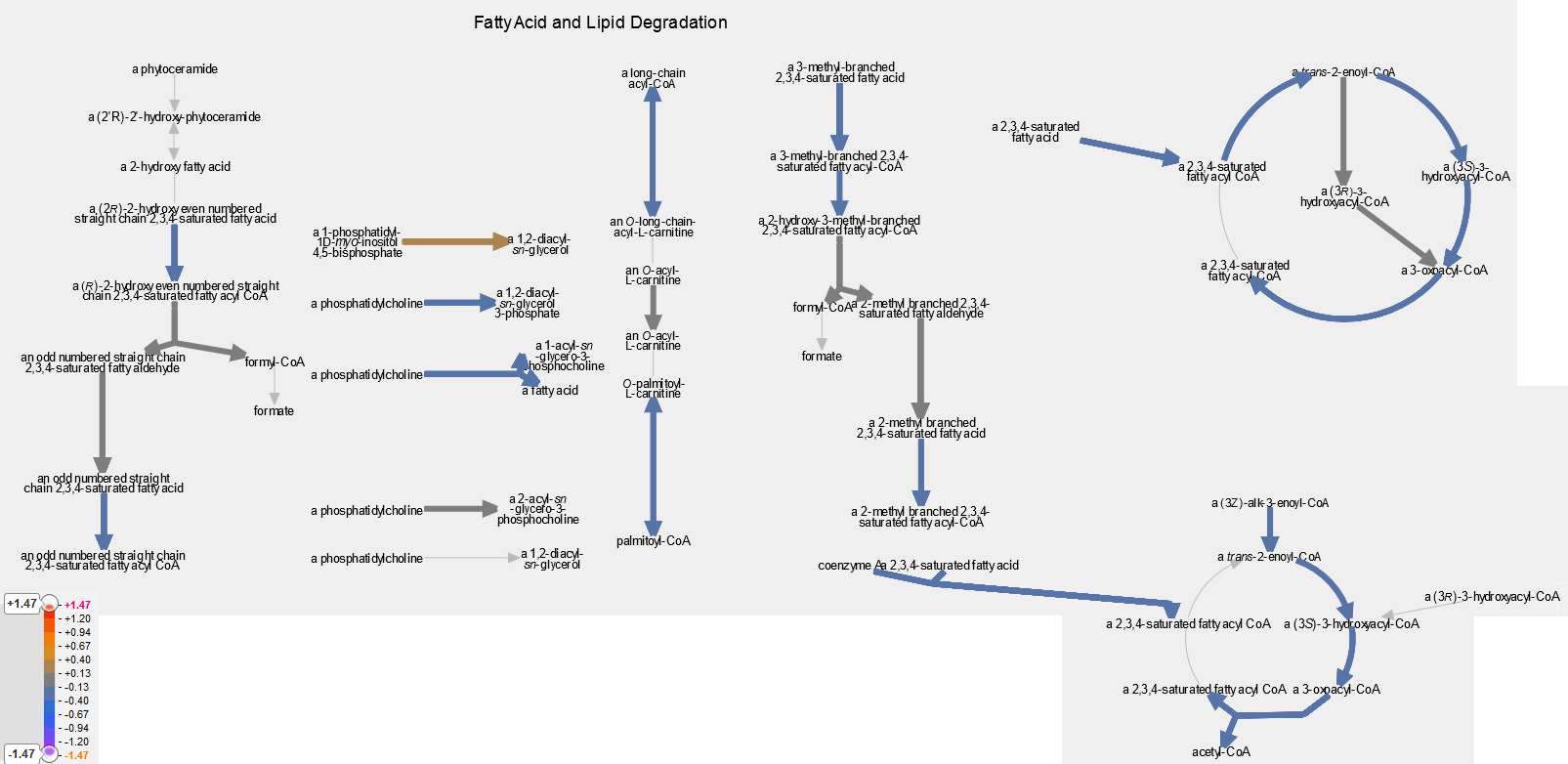


SuppPathway1: Protein abundance changes in selected fatty acid and lipid degradation pathways. Arrow colors indicate the Log ratios UC/healthy according to the color scale. Generated with https://humancyc.org.[1]


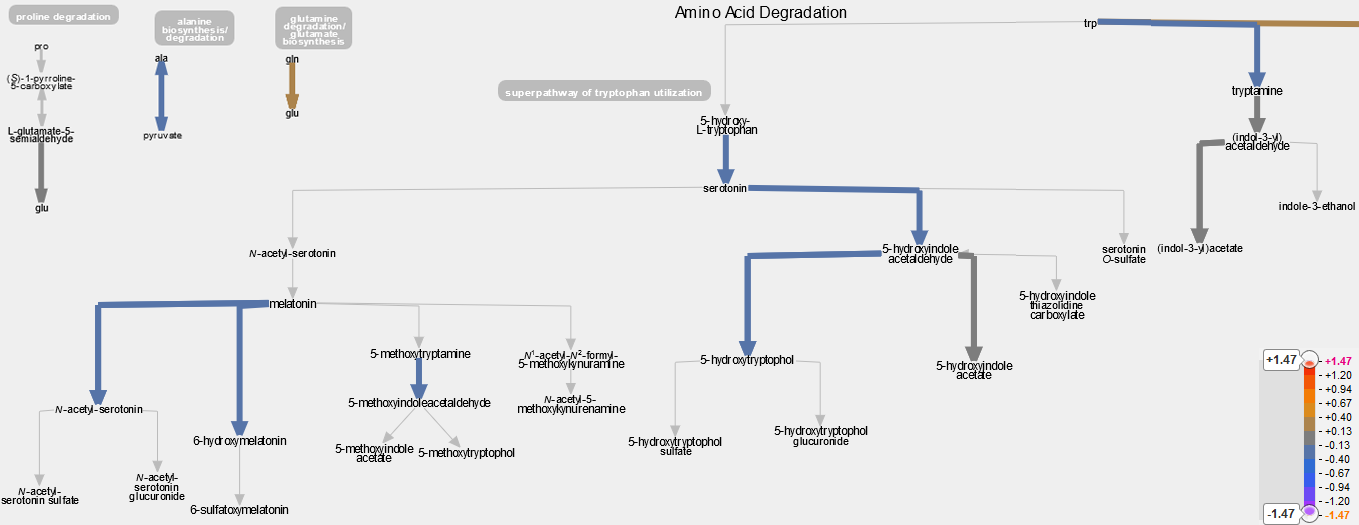


SuppPathway2: Protein abundance changes in selected amino acid degradation pathways (part 1), with focus on tryptophan (trp). Arrow colors indicate the Log ratios UC/healthy according to the color scale. Generated with https://humancyc.org.[1]


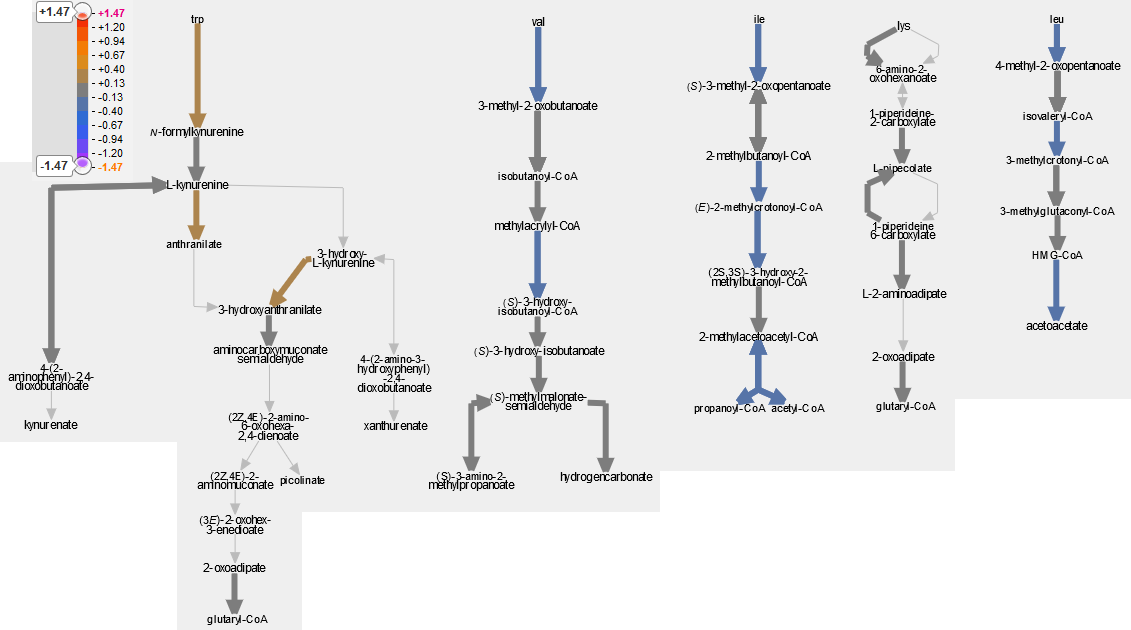


SuppPathway3: Protein abundance changes in selected amino acid degradation pathways (part 2). Arrow colors indicate the Log ratios UC/healthy according to the color scale. Generated with https://humancyc.org.[1]


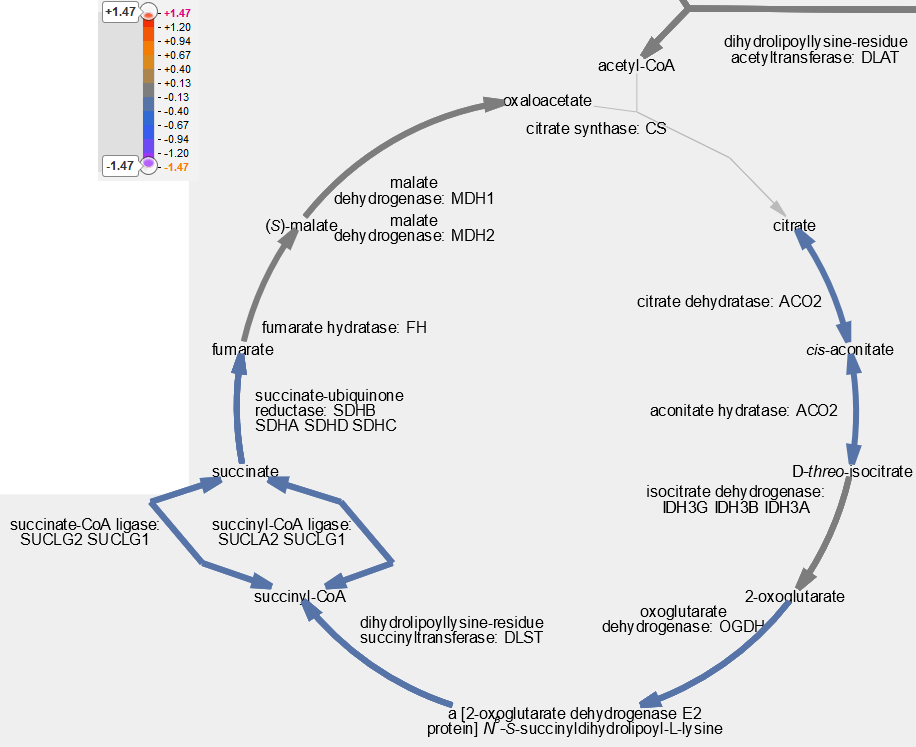


SuppPathway4: Protein abundance changes in tricarboxylic acid (TCA) cycle. Arrow colors indicate the Log ratios UC/healthy according to the color scale. Generated with https://humancyc.org.[1]


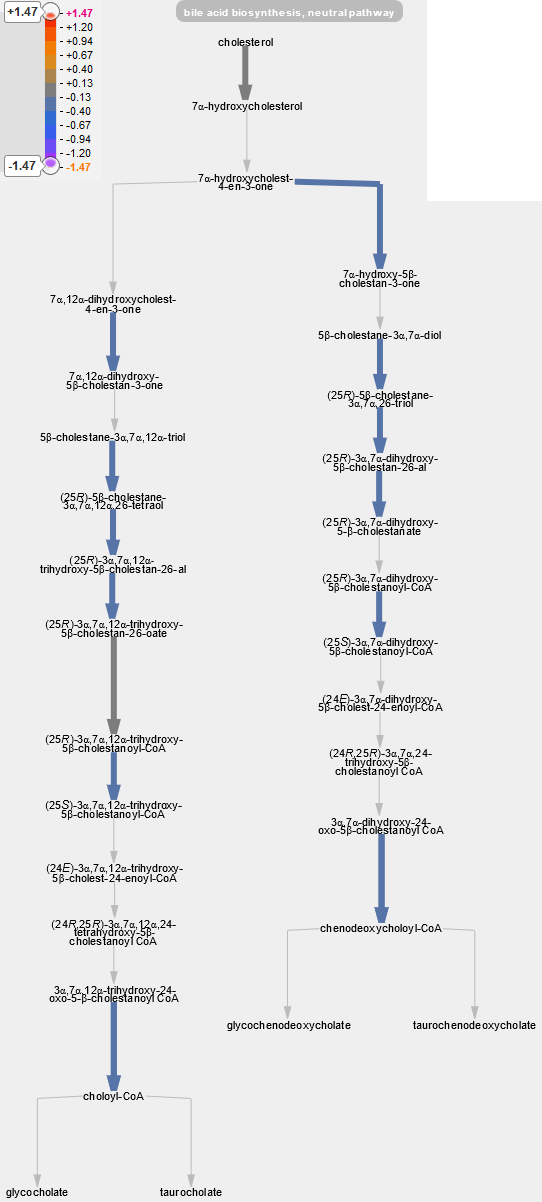


SuppPathway5: Protein abundance changes in bile acid synthesis, neutral pathway. Arrow colors indicate the Log ratios UC/healthy according to the color scale. Generated with https://humancyc.org.[1]

1. Romero P, Wagg J, Green ML, Kaiser D, Krummenacker M, Karp PDJGB: **Computational prediction of human metabolic pathways from the complete human genome**. 2004, **6**(1):R2.
